# Supplementary material for: MetaRibo-Seq measures translation in microbiomes
Source: Nat Commun. 2020 Jun 29;11:3268. doi: 10.1038/s41467-020-17081-z (PMC7324362; doi:10.1038/s41467-020-17081-z)
Supplement: Supplementary file 10 — Supplementary Data 7 [file 41467_2020_17081_MOESM10_ESM.zip › File2/Confidence_High_Taxonomy/31225_out.krona.html]

Javascript must be enabled to view this page.

members
magnitude
magnitudeUnassigned
count
unassigned
taxon
rank

31225\_out

293

293
2
superkingdom


SRS013521\_contig\_number\_contig-100\_36138.36138SRS016954\_contig\_number\_contig-100\_1407.161103SRS042628\_contig\_number\_contig-100\_3539.207142SRS1041038\_contig\_number\_contig-100\_5644.105788SRS144506\_contig\_number\_contig-100\_7112.319834SRS147271\_contig\_number\_contig-100\_1920.246039SRS893383\_contig\_number\_contig-100\_6890.211016
1239
phylum
293
7

1
2292897

SRS1041133\_contig\_number\_contig-100\_579.154891
species

class
277
186801


SRS013951\_contig\_number\_contig-100\_953.240134SRS015782\_contig\_number\_27869SRS017191\_contig\_number\_contig-100\_4920.129237SRS020869\_contig\_number\_contig-100\_6356.234933SRS050422\_contig\_number\_contig-100\_2497.204401SRS1041091\_contig\_number\_contig-100\_4904.129931
186802
order
6
277

541000
32
family


SRS019397\_contig\_number\_8095SRS049402\_contig\_number\_contig-100\_1183.117905SRS055982\_contig\_number\_19023SRS101433\_contig\_number\_contig-100\_1488.111113SRS148511\_contig\_number\_contig-100\_1477.138873
5
1898205
species


SRS077552\_contig\_number\_contig-100\_1030.176432
1263
genus
1
17

species

SRS017916\_contig\_number\_contig-100\_366.121254
2302958
1


SRS015782\_contig\_number\_39027
2293184
1
species

species
1
1262953

SRS147139\_contig\_number\_contig-100\_630.248979

species

SRS048060\_contig\_number\_13782
1
46228


SRS011134\_contig\_number\_contig-100\_1960.307797SRS014855\_contig\_number\_contig-100\_612.109400SRS015578\_contig\_number\_36826SRS017701\_contig\_number\_19698SRS049995\_contig\_number\_31466SRS104084\_contig\_number\_11608SRS143181\_contig\_number\_contig-100\_19261.19261SRS147346\_contig\_number\_1722SRS893230\_contig\_number\_contig-100\_1107.81910
9
165186
species

2293158
3

SRS063127\_contig\_number\_contig-100\_2439.41969SRS077849\_contig\_number\_contig-100\_1325.199575SRS147614\_contig\_number\_contig-100\_5215.73426
species

9
1
genus
216851

SRS011134\_contig\_number\_43739


SRS014412\_contig\_number\_contig-100\_441.123693SRS018623\_contig\_number\_1122SRS019445\_contig\_number\_24346SRS019496\_contig\_number\_contig-100\_836.113726SRS023715\_contig\_number\_28408SRS053214\_contig\_number\_contig-100\_2915.133670SRS065504\_contig\_number\_24856SRS076756\_contig\_number\_22200
853
8
species

1892380
1
genus

1
1849041

SRS077392\_contig\_number\_contig-100\_6689.262781
species

species

SRS018656\_contig\_number\_72
1
1897043

species
245014
1

SRS016095\_contig\_number\_contig-100\_7186.45442

family
132
31979

genus
131
70

SRS011134\_contig\_number\_contig-100\_1972.307809SRS012849\_contig\_number\_contig-100\_1303.212365SRS013476\_contig\_number\_17512SRS013940\_contig\_number\_contig-100\_2749.135844SRS013965\_contig\_number\_42524SRS014235\_contig\_number\_contig-100\_2031.277609SRS014855\_contig\_number\_contig-100\_1095.109883SRS015579\_contig\_number\_15547SRS015663\_contig\_number\_contig-100\_2313.215288SRS015890\_contig\_number\_contig-100\_2326.148719SRS016989\_contig\_number\_contig-100\_1449.102793SRS018817\_contig\_number\_contig-100\_3989.150510SRS019030\_contig\_number\_contig-100\_1741.120755SRS019685\_contig\_number\_contig-100\_881.231765SRS023583\_contig\_number\_contig-100\_1077.106692SRS024075\_contig\_number\_contig-100\_1153.110039SRS024132\_contig\_number\_contig-100\_1394.296650SRS024132\_contig\_number\_contig-100\_6875.302131SRS024331\_contig\_number\_contig-100\_523.209098SRS024435\_contig\_number\_contig-100\_3216.255251SRS024435\_contig\_number\_contig-100\_4023.256058SRS042284\_contig\_number\_contig-100\_1370.52831SRS043001\_contig\_number\_8758SRS043411\_contig\_number\_contig-100\_1202.110114SRS043841\_contig\_number\_10783SRS048870\_contig\_number\_contig-100\_2396.141514SRS050752\_contig\_number\_contig-100\_6345.179723SRS050998\_contig\_number\_contig-100\_1434.103123SRS052027\_contig\_number\_contig-100\_1547.123341SRS053573\_contig\_number\_2147SRS056259\_contig\_number\_23492SRS058070\_contig\_number\_contig-100\_1504.190057SRS063040\_contig\_number\_contig-100\_910.910SRS063040\_contig\_number\_contig-100\_8498.49000SRS064645\_contig\_number\_1903SRS075341\_contig\_number\_contig-100\_650.158615SRS075984\_contig\_number\_27991SRS075984\_contig\_number\_27991SRS076929\_contig\_number\_contig-100\_2019.2019SRS076976\_contig\_number\_7102SRS077294\_contig\_number\_contig-100\_45353.45353SRS078176\_contig\_number\_contig-100\_1570.144095SRS078419\_contig\_number\_37978SRS097920\_contig\_number\_contig-100\_1340.194120SRS098073\_contig\_number\_contig-100\_952.120372SRS1041037\_contig\_number\_contig-100\_4719.98808SRS1041134\_contig\_number\_contig-100\_4477.89322SRS1041145\_contig\_number\_contig-100\_1543.197561SRS105153\_contig\_number\_contig-100\_7441.196853SRS142542\_contig\_number\_contig-100\_2963.168605SRS142542\_contig\_number\_contig-100\_2963.168605SRS142781\_contig\_number\_10433SRS143148\_contig\_number\_contig-100\_1001.159496SRS143148\_contig\_number\_contig-100\_2857.161352SRS143372\_contig\_number\_5778SRS143598\_contig\_number\_contig-100\_4330.122313SRS143722\_contig\_number\_contig-100\_1002.94469SRS144183\_contig\_number\_7398SRS146764\_contig\_number\_contig-100\_1280.207645SRS147425\_contig\_number\_contig-100\_17804.58102SRS148319\_contig\_number\_6446SRS148721\_contig\_number\_contig-100\_1971.314607SRS148784\_contig\_number\_contig-100\_1262.169350SRS148784\_contig\_number\_contig-100\_4772.172860SRS148817\_contig\_number\_contig-100\_1381.89988SRS148817\_contig\_number\_contig-100\_1381.89988SRS893230\_contig\_number\_contig-100\_1080.81883SRS893295\_contig\_number\_contig-100\_2606.85230SRS893295\_contig\_number\_contig-100\_2606.85230SRS893373\_contig\_number\_contig-100\_537.156908
1485

species
2293011
1

SRS893187\_contig\_number\_13212

species

SRS019161\_contig\_number\_32997
411489
1


SRS020869\_contig\_number\_32511
1262781
1
species


SRS015578\_contig\_number\_contig-100\_5995.182433SRS063985\_contig\_number\_15718SRS148159\_contig\_number\_16716
3
2292206
species


SRS019496\_contig\_number\_contig-100\_998.113888SRS075773\_contig\_number\_30547SRS1041136\_contig\_number\_contig-100\_1752.147476
2293016
3
species

59620
47

SRS012849\_contig\_number\_contig-100\_2559.207970SRS013687\_contig\_number\_contig-100\_343.197625SRS013951\_contig\_number\_contig-100\_17658.66017SRS014613\_contig\_number\_contig-100\_1184.130113SRS015431\_contig\_number\_30408SRS015431\_contig\_number\_73252SRS015782\_contig\_number\_contig-100\_1811.261873SRS016018\_contig\_number\_7284SRS016335\_contig\_number\_49634SRS017191\_contig\_number\_contig-100\_1115.45833SRS017307\_contig\_number\_contig-100\_1571.183111SRS017433\_contig\_number\_contig-100\_2772.72128SRS017821\_contig\_number\_contig-100\_775.195675SRS019068\_contig\_number\_15399SRS020233\_contig\_number\_contig-100\_1669.344016SRS023914\_contig\_number\_contig-100\_2579.167225SRS042628\_contig\_number\_24329SRS043768\_contig\_number\_23223SRS045826\_contig\_number\_contig-100\_2192.131557SRS047044\_contig\_number\_contig-100\_1670.102994SRS049896\_contig\_number\_contig-100\_3573.207603SRS052697\_contig\_number\_contig-100\_4442.263272SRS053214\_contig\_number\_contig-100\_3347.3348SRS054905\_contig\_number\_contig-100\_614.147066SRS063040\_contig\_number\_21854SRS063190\_contig\_number\_297SRS063518\_contig\_number\_contig-100\_2546.20291SRS064757\_contig\_number\_17152SRS076929\_contig\_number\_18217SRS077194\_contig\_number\_contig-100\_1448.193468SRS077730\_contig\_number\_contig-100\_1113.43765SRS098571\_contig\_number\_40248SRS1041142\_contig\_number\_contig-100\_505.62732SRS104975\_contig\_number\_contig-100\_26727.26728SRS1055038\_contig\_number\_contig-100\_6039.6040SRS1055067\_contig\_number\_16219SRS142503\_contig\_number\_contig-100\_5849.337443SRS142923\_contig\_number\_contig-100\_961.138029SRS143342\_contig\_number\_contig-100\_3215.141427SRS144506\_contig\_number\_42281SRS146764\_contig\_number\_contig-100\_2239.208604SRS147346\_contig\_number\_contig-100\_4155.359967SRS148319\_contig\_number\_15313SRS893270\_contig\_number\_14021SRS893292\_contig\_number\_contig-100\_13643.47750SRS893378\_contig\_number\_contig-100\_716.143734SRS893383\_contig\_number\_29572
species

1262802
4

SRS049446\_contig\_number\_contig-100\_4608.97646SRS056259\_contig\_number\_contig-100\_3101.173824SRS063985\_contig\_number\_25176SRS142503\_contig\_number\_43180
species

species

SRS051031\_contig\_number\_contig-100\_390.205573
2293052
1


SRS013476\_contig\_number\_contig-100\_1307.230486
1
2292288
species

2
94
family
186803

SRS021948\_contig\_number\_contig-100\_764.207083SRS971275\_contig\_number\_contig-100\_4379.282038

genus
2569097
1

species

SRS077502\_contig\_number\_contig-100\_346.117982
39488
1

genus
3
1
189330

SRS146813\_contig\_number\_contig-100\_4687.167156


SRS023914\_contig\_number\_contig-100\_7688.7688SRS147022\_contig\_number\_6135
2
39486
species

genus
1506577
1

29361
1

SRS144537\_contig\_number\_contig-100\_2107.252429
species

841

SRS013800\_contig\_number\_contig-100\_958.161189SRS893295\_contig\_number\_contig-100\_1429.84053
genus
2
23

species

SRS016095\_contig\_number\_contig-100\_3145.173701SRS024625\_contig\_number\_contig-100\_26972.54928SRS046369\_contig\_number\_contig-100\_3142.44795SRS063040\_contig\_number\_43701SRS097889\_contig\_number\_contig-100\_2817.230471SRS104693\_contig\_number\_2837SRS1055069\_contig\_number\_contig-100\_447.100395SRS143070\_contig\_number\_contig-100\_3501.120076SRS148159\_contig\_number\_contig-100\_5825.336599SRS893342\_contig\_number\_contig-100\_1743.72204
360807
10

species

SRS015854\_contig\_number\_24829SRS045244\_contig\_number\_4358SRS051031\_contig\_number\_contig-100\_621.205804
3
301301

6
166486

SRS016954\_contig\_number\_contig-100\_785.160481SRS077641\_contig\_number\_2898SRS098827\_contig\_number\_9593SRS103987\_contig\_number\_37805SRS1055034\_contig\_number\_13668SRS148721\_contig\_number\_23370
species

species
2293138
1

SRS1055034\_contig\_number\_10766

2049040
1

SRS024132\_contig\_number\_46763
species

species

SRS053398\_contig\_number\_contig-100\_316.163450SRS149784\_contig\_number\_contig-100\_502.167104
2109691
2

genus
1
17

SRS144603\_contig\_number\_contig-100\_1174.104808
572511


SRS044535\_contig\_number\_contig-100\_1401.148332SRS149181\_contig\_number\_contig-100\_1520.186799
2
33038
species


SRS104036\_contig\_number\_1530
1
2292961
species

species
2292963
5

SRS014923\_contig\_number\_contig-100\_1173.247650SRS018313\_contig\_number\_contig-100\_1397.87551SRS049900\_contig\_number\_contig-100\_132.126508SRS054956\_contig\_number\_contig-100\_1293.97947SRS076929\_contig\_number\_27076

33039
2

SRS050941\_contig\_number\_16833SRS144362\_contig\_number\_contig-100\_867.260517
species

species

SRS018427\_contig\_number\_contig-100\_1779.187943SRS023526\_contig\_number\_contig-100\_2012.231665SRS104311\_contig\_number\_contig-100\_353.266330SRS1055049\_contig\_number\_contig-100\_1901.99069
4
40520


SRS024331\_contig\_number\_25844
1
1917876
species

species

SRS150029\_contig\_number\_contig-100\_1689.127506
1805476
1


SRS013638\_contig\_number\_contig-100\_31574.31574SRS049959\_contig\_number\_6033
33042
genus
2
9


SRS147557\_contig\_number\_13394
116085
1
species

1
1896998

SRS147377\_contig\_number\_12063
species

species

SRS015190\_contig\_number\_14059
2293096
1

species
4
410072

SRS018936\_contig\_number\_contig-100\_764.92573SRS022071\_contig\_number\_contig-100\_1029.233316SRS045713\_contig\_number\_contig-100\_6759.129989SRS104400\_contig\_number\_contig-100\_1647.347245

28050
4
genus

species
28052
4

SRS015431\_contig\_number\_contig-100\_16251.197545SRS047044\_contig\_number\_contig-100\_2344.103668SRS058723\_contig\_number\_contig-100\_942.64177SRS147919\_contig\_number\_contig-100\_3609.124635


SRS019030\_contig\_number\_contig-100\_1210.120224
39491
1
species

species
31
1898203

SRS011239\_contig\_number\_20057SRS014923\_contig\_number\_26796SRS015065\_contig\_number\_39373SRS016495\_contig\_number\_13095SRS017916\_contig\_number\_9118SRS019068\_contig\_number\_56015SRS019161\_contig\_number\_43246SRS019496\_contig\_number\_3960SRS019808\_contig\_number\_18387SRS022609\_contig\_number\_44023SRS024492\_contig\_number\_18375SRS053335\_contig\_number\_15071SRS054352\_contig\_number\_24173SRS063985\_contig\_number\_19622SRS064757\_contig\_number\_18625SRS077024\_contig\_number\_contig-100\_3384.101304SRS098644\_contig\_number\_contig-100\_3885.283322SRS098881\_contig\_number\_26539SRS1041038\_contig\_number\_15015SRS1041136\_contig\_number\_14981SRS104975\_contig\_number\_248SRS140513\_contig\_number\_15910SRS142503\_contig\_number\_19950SRS143895\_contig\_number\_contig-100\_6403.247044SRS144506\_contig\_number\_57985SRS147271\_contig\_number\_13365SRS147766\_contig\_number\_8042SRS149784\_contig\_number\_contig-100\_3658.170260SRS893270\_contig\_number\_contig-100\_4220.138653SRS893358\_contig\_number\_contig-100\_30026.30027SRS971276\_contig\_number\_20777

10
186806
family

1730

SRS020233\_contig\_number\_47690
1
10
genus

species
2
1262887

SRS014313\_contig\_number\_contig-100\_2429.55010SRS077335\_contig\_number\_9622

species
4
39485

SRS019601\_contig\_number\_2077SRS019693\_contig\_number\_9093SRS052697\_contig\_number\_46714SRS098073\_contig\_number\_19470

species
1
39490

SRS045645\_contig\_number\_21327


SRS100021\_contig\_number\_12408SRS145497\_contig\_number\_14571
2
142586
species

family
216572
1

genus
1
459786

1945593
1

SRS075821\_contig\_number\_6362
species

species
2292887
1

SRS053356\_contig\_number\_contig-100\_1759.276725

526524
1
class

526525
1
order

family
1
128827

1
1505663
genus

species

SRS075078\_contig\_number\_32139
1547
1

1262994
1

SRS017307\_contig\_number\_contig-100\_2018.183558
species

2292899
1

SRS047433\_contig\_number\_contig-100\_1099.134034
species

3
1897036

SRS018836\_contig\_number\_contig-100\_575.167258SRS043768\_contig\_number\_contig-100\_1897.194030SRS077730\_contig\_number\_2576
species

1897027
1

SRS047433\_contig\_number\_contig-100\_662.38012
species
